# Post-COVID-19 Syndrome: Retinal Microcirculation as a Potential Marker for Chronic Fatigue

**DOI:** 10.3390/ijms232213683

**Published:** 2022-11-08

**Authors:** Sarah Schlick, Marianna Lucio, Gerd Wallukat, Alexander Bartsch, Adam Skornia, Jakob Hoffmanns, Charlotte Szewczykowski, Thora Schröder, Franziska Raith, Lennart Rogge, Felix Heltmann, Michael Moritz, Lorenz Beitlich, Julia Schottenhamml, Martin Herrmann, Thomas Harrer, Marion Ganslmayer, Friedrich E. Kruse, Robert Lämmer, Christian Mardin, Bettina Hohberger

**Affiliations:** 1 Department of Ophthalmology, Universitätsklinikum Erlangen, Friedrich-Alexander-Universität Erlangen-Nürnberg, 91054 Erlangen, Germany; 2 Research Unit Analytical BioGeoChemistry, Helmholtz Zentrum München-German Research Center for Environmental Health, 85764 Neuherberg, Germany; 3 Berlin Cures GmbH, 10719 Berlin, Germany; 4 Department of Internal Medicine 3, Universitätsklinikum Erlangen, Friedrich-Alexander-Universität Erlangen-Nürnberg, 91054 Erlangen, Germany; 5 Deutsches Zentrum für Immuntherapie (DZI), Friedrich-Alexander-Universität Erlangen-Nürnberg (FAU) and Universitätsklinikum Erlangen, 91054 Erlangen, Germany; 6 Department of Internal Medicine 1, Universität of Erlangen-Nürnberg, Friedrich-Alexander-Universität Erlangen-Nürnberg, 91054 Erlangen, Germany

**Keywords:** retinal microcirculation, post-COVID-19 syndrome, chronic fatigue, functional GPCR autoantibodies, COVID-19, long COVID syndrome, chronic fatigue syndrome, OCT angiography

## Abstract

Post-COVID-19 syndrome (PCS) is characterized by persisting sequelae after infection with severe acute respiratory syndrome coronavirus 2 (SARS-CoV-2). PCS can affect patients with all COVID-19 disease severities. As previous studies have revealed impaired blood flow as a provoking factor triggering PCS, it was the aim of the present study to investigate the potential association between self-reported chronic fatigue and retinal microcirculation in patients with PCS, potentially indicating an objective biomarker. A prospective study was performed, including 201 subjects: 173 patients with PCS and 28 controls. Retinal microcirculation was visualized by OCT angiography (OCT-A) and quantified using the Erlangen-Angio-Tool as macula and peripapillary vessel density (VD). Chronic fatigue (CF) was assessed according to the variables of Bell’s score, age and gender. VDs in the superficial vascular plexus (SVP), intermediate capillary plexus (ICP) and deep capillary plexus (DCP) were analyzed, considering the repetitions (12 times). Seropositivity for autoantibodies targeting G protein-coupled receptors (GPCR-AAbs) was determined by an established cardiomyocyte bioassay. Taking account of the repetitions, a mixed model was performed to detect possible differences in the least square means between the different groups included in the analysis. An age effect in relation to VD was observed between patients and controls (*p* < 0.0001). Gender analysis showed that women with PCS showed lower VD levels in the SVP compared to male patients (*p* = 0.0015). The PCS patients showed significantly lower VDs in the ICP as compared to the controls (*p* = 0.0001 (CI: 0.32; 1)). Moreover, considering PCS patients, the mixed model revealed a significant difference between those with chronic fatigue (CF) and those without CF with respect to VDs in the SVP (*p* = 0.0033 (CI: −4.5; −0.92)). The model included variables of age, gender and Bell’s score, representing a subjective marker for CF. Consequently, retinal microcirculation might serve as an objective biomarker in subjectively reported chronic fatigue in patients with PCS.

## 1. Introduction

Severe acute respiratory syndrome coronavirus 2 (SARS-CoV-2), first observed in Wuhan, Hubei Province, China, in 2019 [1], was declared a public health emergency of international concern in January 2020 by the World Health Organization (WHO) [2]. In March 2020, worldwide pandemic infection levels were reached, with impacts on social life, the economy, and healthcare systems [3]. Acute coronavirus disease 2019 (COVID-19) caused a number of pneumonia cases [1] and can lead to various complications, such as respiratory and multiorgan failure [3]. By 18 February 2022, the virus’s spread had increased to over 418 million cases, with over 5.8 million deaths (numbers obtained from the WHO) [4,5]. Apart from acute COVID-19 disease, post-COVID-19 syndrome (PCS) can arise afterwards.

PCS is defined as the persistence of symptoms for more than 12 weeks after infection with the virus (S1 guideline; AWMF online). Continuing symptoms more than 4 weeks after infection are recognized as long COVID symptoms or post-acute sequelae of COVID-19 [6]. 

The most common symptoms reported in studies are chronic fatigue (CF) and dyspnea (i.e., shortness of breath) [5]. Persistent symptoms may include neurocognitive impairments (brain fog, loss of attention), autonomic symptoms (chest pain, palpitations, tachycardia), gastrointestinal issues, musculoskeletal problems (myalgia), smell and taste dysfunction, cough, headache and hair loss [5,6,7]. Studies have reported that PCS can even affect people with moderately acute COVID-19 who did not require hospital care during the acute stage [5,8,9]. The absolute numbers of patients with PCS correspond to the shape and amplitude of the pandemic curve, showing the risk PCS poses for individual health, healthcare systems and the economy [10]. Studies have revealed that more than half of patients with COVID-19 infection reported PCS symptoms [11]. Others have postulated that 15% of all COVID-19 patients [6] and 50–70% of hospitalized patients suffer from PCS [7].

The pathogenesis of PCS is still elusive. Recent studies have revealed viral persistence and enduring texture damage, including endotheliopathy, impaired microvasculature, hypercoagulation, thrombosis, neutrophil extracellular traps (NETs), chronic immune dysregulation, dysregulation of the renin–angiotensin–aldosterone system (RAAS) and hyperinflammation/autoimmunity, as possible pathomechanisms of PCS [6,12,13,14]. It is assumed that autoimmunphenomena [15], including the generation of functional active autoantibodies [5,16], are involved in the pathogenesis of PCS with potential different PCS subgroups [5,6]. Autoantibodies targeting G protein-coupled receptors (GPCR-AAbs) are of especial interest, as GPCRs represent the largest receptor family in humans. A functional dysbalance in these receptors, induced by functional active GPCR-AAbs, is likely to disturb several factors in the human body. A previous study in GPCR-AAb-positive glaucoma patients having shown a link with impaired microcirculation [17], an experimental therapy aiming to neutralize functional active GPCR-AAbs improved PCS in a glaucoma patient [16]. Thus, it can be hypothesized that GPCR-AAbs are involved in the pathogenesis of PCS, potentially in combination with preconditions (e.g., ischemia) [5,18,19]. In addition, recent studies have examined increased D-dimer levels up to 4 months post-acute infection in approximately 25% of patients [20]. The mechanisms of these persistent procoagulant effects in PCS have not been clarified at this point in time [20]. Endotheliopathy and elevated plasma markers of endothelial cell activation have been recognized in patients with severe COVID-19 [20]. Studies have investigated persistent endotheliopathy in patients with PCS which is associated with enhanced thrombin generation potential independently of ongoing acute-phase response or NETosis [20]. Autopsy studies revealed that alveolar capillary microthrombi were nine times more prevalent in patients with COVID-19 compared to patients with influenza [21]. It can be assumed that impaired microcirculation might be one factor contributing to the clinical symptoms of PCS.

Fatigue is one of the most common symptoms of PCS, which emphasizes its impact on individual health, healthcare systems and economics [6,11,22]. The study “Assessment and characterization of post-COVID-19 manifestations” revealed that fatigue was reported as the most common symptom in 72.8% of patients [23]. Sudre et al. related data from the COVID-19 symptom study app and concluded that “self-reported fatigue is the commonest complaint in a large group of Long-COVID patients” [24,25]. Associations between fatigue in PCS and laboratory markers of inflammation and cell turnover (leukocyte, neutrophil or lymphocyte counts; neutrophil-to-lymphocyte ratio; lactate dehydrogenase and C-reactive protein levels) or pro-inflammatory molecules (IL-6 or sCD25) had not been observed until recently [11]. Thus, it would be of interest to establish an objective marker for patient self-reported fatigue.

The eye, as a window into the human body, can be used as a “diagnostic window” for several systemic disorders. The retinal capillary system represents the microcirculation in the whole human body; thus, retinal capillary disorders might represent whole human microcirculatory disorders. Retinal macular and peripapillary capillary plexuses can be visualized by optical coherence tomography angiography (OCT-A) and quantified using the Erlangen-Angio-Tool (EA-Tool) [5,26,27,28]. OCT-A is easy to perform and allows for non-invasive measurement without any contact with the human eye. It measures differences in the speckle patterns of backscattered light in two or more repeated scans. These differences are caused by moving particles, such as red blood cells (RBCs) [29]. The aim of this study was to investigate the association between self-reported chronic fatigue and retinal microcirculation in patients with PCS to potentially indicate an objective biomarker. In addition, serum samples were screened for GPCR-AAbs, considering their potential impact on microcirculation.

## 2. Results

LS means of overall VD were 29.97 ± 0.06 (SVP), 21.96 ± 0.05 (ICP) and 23.62 ± 0.06 (DCP) in patients with PCS. Controls showed LS means of overall VD of 30.13 ± 0.19 (SVP), 22.62 ± 0.17 (ICP) and 23.73 ± 0.19 (DCP). 

**Figure 1:**
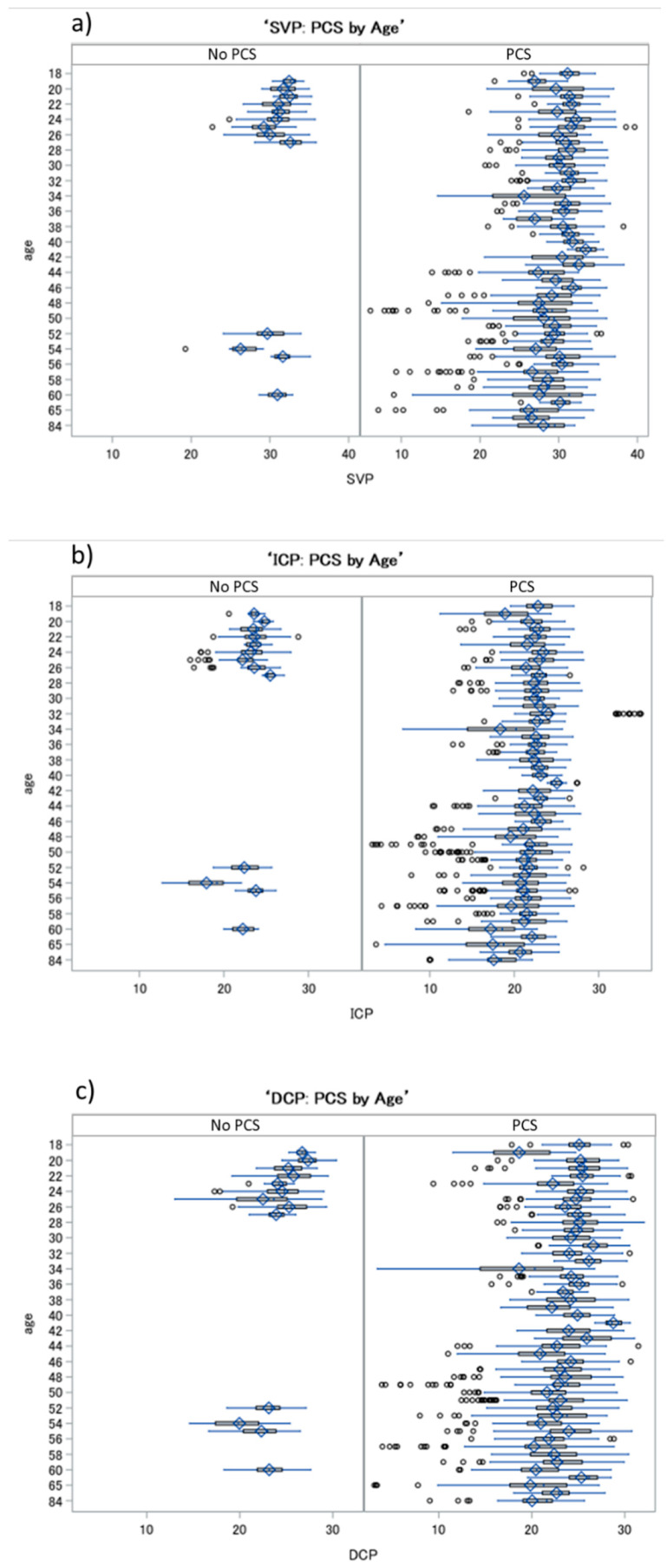
Boxplots of VDs in the SVP (**a**), ICP (**b**) and DCP (**c**) in patients with post-COVID-19 syndrome (PCS) and controls, considering the cofactor of age: a decrease in VD in all retinal layers was observed with increasing age in PCS patients and controls. SVP—superficial vascular plexus, ICP—intermediate capillary plexus, DCP—deep capillary plexus.

Significant effects for age and gender were observed with respect to VD in the SVP, ICP and DCP (*p* < 0.0001). Considering the influence of age, we observed that, with increasing age, VD in the SVP, ICP and DCP decreased (Figure 1). Estimated values were −0.06 (SVP), −0.06 (ICP) and −0.07 (DCP) in patients with PCS.

**Figure 2:**
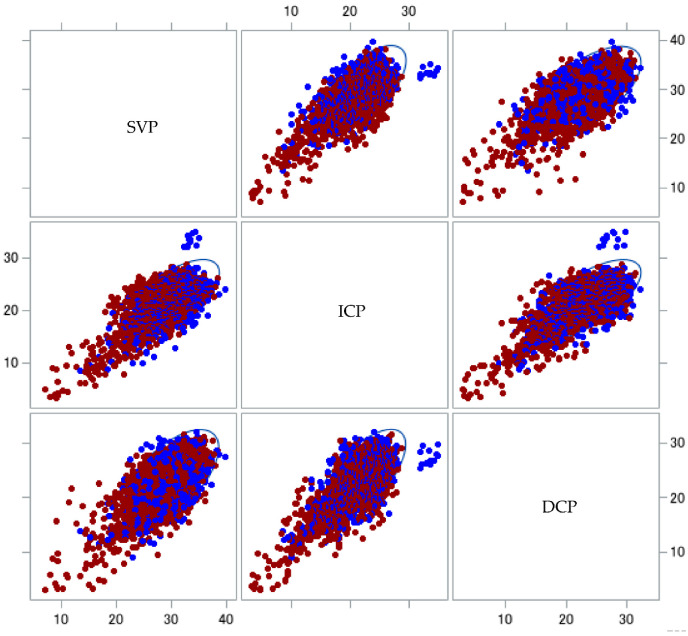
Scatter plot of vessel density differences in patients with post-COVID-19 syndrome (PCS) in relation to gender: matrix showing the relationship between vessel densities in the SVPs, ICPs and DCPs of patients with PCS. Each set of vessel density data is color-coded according to gender (male, blue; female, red), with 95% prediction ellipses. Female patients with PCS and CF showed lower VDs. SVP—superficial vascular layer, ICP—intermediate capillary plexus, DCP—deep capillary plexus.

**Figure 3:**
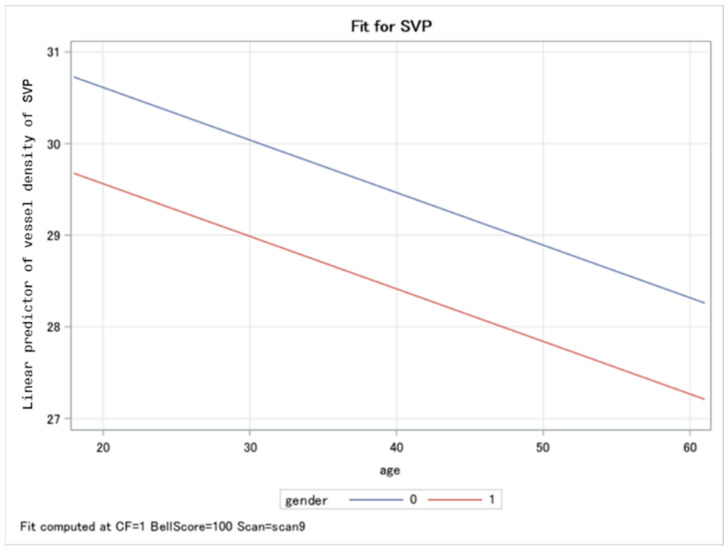
Panel plot overlaying the predicted values for male (blue line) and female (red line) vessel densities in the superficial vascular layer (SVP) among patients with post-COVID-19 symptoms: women patients showed significantly lower VDs in the SVP compared to men with increasing age.

Correlations between gender and VD in the SVP, ICP and DCP in patients with PCS are plotted in Figure 2. Females showed lower levels for each comparison of retinal layers. Significantly lower (Type 3 Tests of Fixed Effects) VDs in the SVP in female patients with PCS were observed compared to males (LS mean difference = 1.05 (CI: 0.41; 1.69), *p* = 0.0015; Figure 3) with increasing age.

The analysis using a mixed model with 12 repetitions, which was corrected for age and gender, yielded significantly impaired VDs in the ICP (*p* = 0.0001), yet not in the SVP or DCP (*p* > 0.05) in patients with PCS, compared to controls (Table 1).

Instead, in the PCS patients, the complete model (including age, gender and Bell’s score variables) revealed a significant difference between patients with chronic fatigue (CF) and those without CF with respect to VD in the SVP (*p* = 0.0033, (CI: −4.5; −0.92)) (Table 2). No notable differences were observed for the other retinal layers (*p* > 0.05). Patients with PCS and CF showed LS means of VD of 30.3 ± 0.28 (SVP), 21.89 ± 0.25 (ICP) and 23.08 ± 0.24 (DCP). Patients with PCS without CF showed LS means of VD of 27.59 ± 0.91 (SVP), 21.58 ± 0.84 (ICP) and 23.69 ± 0.8 (DCP).

Considering GPCR-AAbs and their potential impact on microcirculation in patients with PCS, a mixed model with repetitions was generated, with combinations of GPCR-AAbs inserted. Significant effects of β2-AAb on VD in the SVP (*p* = 0.0075), Noci-AAb (*p* = 0.0344) and β2-AAb (*p* = 0.0112) and of ETA-AAb (0.0261) on VD in the ICP, along with a trend associating Noci-AAb (*p* = 0.055) with VD in the DVP, were observed in the present cohort (Table 3).

## 3. Discussion

Post-COVID-19 syndrome (PCS) is a challenge for individual health, the healthcare system and the economy due to its high prevalence in patients worldwide. The leading clinical symptom of PCS is self-reported fatigue, as it is one of the most common symptoms associated with PCS among large groups of patients [6,11,22,23,24,25]. The prevalence of CF is not associated with COVID-19 severity, which indicates that it potentially affects a high number of patients [11]. As each clinician prefers objective biomarkers in addition to self-reported clinical symptoms, the aim of this study was to investigate the association of self-reported chronic fatigue and retinal microcirculation in patients with PCS to potentially indicate an objective biomarker. An age effect with respect to VD was observed in patients and controls (*p* < 0.0001). Gender analysis revealed that women with PCS showed lower VD levels in the SVP especially, compared to male patients (*p* = 0.0015). Previous studies revealed a PCS ratio of 3:1 in women and men [30,31]. In addition, the present data conform with clinical observations that women with PCS had a higher probability of fatigue and anxiety/depression throughout 6-month follow-up [32,33]. Patients with PCS showed significantly lower VDs in the ICP compared to controls (*p* = 0.0001 (CI: 0.32; 1)), considering age and gender effects. Among PCS patients, the mixed model revealed a significant difference between those with chronic fatigue (CF) and those without CF with respect to VDs in the SVP (*p* = 0.0033 (CI: −4.5; −0.92)). The model included as variables age, gender and Bell’s score, the latter representing a subjective marker for CF. The variable of Bell’s score was always significant for each VD. Thus, the eye as a window into the human body might offer an objective diagnostic option through the measurement of retinal microcirculation in cases of self-reported CF among patients with PCS. Considering GPCR-AAb and impaired microcirculation, data for the present cohort showed a significant effect of β2-AAb on VD in the SVP (*p* = 0.0075), Noci-AAb (*p* = 0.0344) and β2-AAb (*p* = 0.0112) and of ETA-AAb (0.0261) on VD in the ICP, along with a trend of association between Noci-AAb (*p* = 0.055) and VD in the DVP.

To date, there is no uniform consensus about the definition of CF in PCS. It is assumed that the label CF/post-COVID-19 fatigue, in line with definitions of post-infectious fatigue, should be applied under the following conditions: that it is a dominant symptom, chronic, disabling (it prevents pre-illness activities and duties), is intensified after mental and/or physical activity (post-exertional malaise, PEM) [34,35], has persisted for 6 months or longer (3 months in children and adolescents), that it occurred during confirmed acute COVID-19 and has persisted without a symptom-free interval since onset [36]. The unknown nature of PCS and its phenotypical similarity to postinfectious fatigue syndrome has led some studies to suggest a connection to myalgic encephalomyelitis/chronic fatigue syndrome (ME/CFS) [37,38,39]. Post-exertional malaise (PEM), a leading symptom of ME/CFS, is characterized by worsening symptoms after low or moderate daily activity for several hours or weeks [35,40]. This burden has also been found in PCS patients [35,40] Patients lose the ability to engage in pre-illness levels of activity in social life, work or school [39]. Studies have revealed that patients react abnormally to stressors, e.g., they wake up with abnormal rises in serum cortisol levels and heart rates [41]. Women seem to be more affected than men [42]. 

CF in PCS exhibits similar incidences in hospitalized and non-hospitalized patients [22,43]. Fatigue and cognitive impairment are assumed to endure and may worsen over time in individuals with <6-month and ≥6-month follow-ups [22,43]. If CF in PCS is identified, there should follow an underlying diagnostic. 

At the moment, CF diagnostics is based on brief questionnaires to characterize the fatigue state, such as the Calder Fatigue Scale or the SPHERE [36]. These methods try to identify CF in line with the disease-specific recommendations from the National Institute of Neurological Disorders and Stroke Common Data Elements [36]. As CF is often part of a multisymptomatic cluster, SPHERE includes related physical symptoms in the diagnostics, and other systems also include mental health questions (e.g., the Patient Health Questionnaire-9) [36]. CF in PCS is associated with marked functional impairment [22]. As a subgroup of patients still exhibited inflammatory markers after acute COVID-19 infection, it has been suggested that hyperinflammation is a cause of CF in PCS [22]. The causal association between specific pro-inflammatory cytokines, mood symptoms and cognitive decline has been confirmed [44,45]. Other post-infectious syndromes (e.g., post-infectious encephalitis) have been associated with inflammatory processes [46]. The pathophysiology of CF remains unresolved [36].

To the best of our knowledge, the present study has revealed for the first time an association between CF in PCS by recourse to Bell’s scores and impaired retinal microcirculation, as determined by OCT-A, potentially indicating an objective biomarker. OCT-A is able to visualize retinal macular and peripapillary capillary plexuses [5,26,27,28]. It is easy to perform and allows for non-invasive measurement without contact with the human eye. The technical basis is the recording of a real-time motion signal based on temporal changes in intravascular moving red blood cells (RBCs) [29]. If a signal is recorded, a retinal pixel is coded ‘white’; without any motion, it is coded by ‘black’ (i.e., the coding is binary) [5]. The data can be analyzed with high reliability and reproducibility using the Erlangen-Angio-Tool (EA-Tool) [33]. Fine-grained analysis can be performed by division of the scan region into 12 sectors (macula) or 4 sectors (peripapillary region) to calculate the overall and sectorial vessel density (VD). The eye, as a “window” into the human body, is representative of several systemic disorders [47,48,49,50,51]. Alveolar capillary occlusion is a characteristic symptom of COVID-19 which can lead in severe cases to respiratory failure, as blood oxygen uptake is limited [22,52]. Impaired microcirculation can be found in acute COVID-19 as well as in PCS [5,53,54,55]. The virus may infect endothelial cells directly via Angiotensin Converting Enzyme 2 (ACE2), leading to inflammation and fibrosis [53], and may trigger the generation of several autoantibodies targeting receptors, being involved in the regulation of blood flow. Specific GPCR-AAbs were observed to have an impact on microcirculation, confirming previous data [5,17,18]. Noci-AAb, β2-AAb and ETA-AAb were observed to be linked to impaired retinal microcirculation in different retinal layers. 

The present study showed that female patients with PCS exhibited lower vessel density (VD) levels in comparisons of their retinal layers (SVPs, ICPs and DCPs) with those of men. This reinforced impaired microcirculation in female patients with PCS is in line with the results of other PCS studies [5]. The analysis, which was corrected for age and gender, showed significantly impaired VD in the ICP in patients with PCS compared to controls. Interestingly, the Angiotensin Converting Enzyme 2 (ACE2), a serine protease, is located in the ICP retinal layer. There is evidence that SARS-CoV-2 occupies the human body by first binding to the ectoenzyme ACE2, acting as the receptor [56]. Another serine protease is required to prime the viral spike “S” protein for entry into cells [56]. Inclusion of the additional explicative variable Bell’s score, representing a subjective marker for CF, in the mixed model, revealed a significant effect on the SVP when comparing PCS patients with CF and without CF. Thus, CF has a significant impact on retinal microcirculation, which indicates that the latter is a potential objective biomarker for CF which might provide an objective diagnostic option for CF in PCS patients. The eye as a window into the human body might offer an objective diagnostic option through the measurement of retinal microcirculation in self-reported chronic fatigue in patients with PCS. It can be assumed that retinal microcirculation can have an impact as a diagnostic tool in PCS patient populations and that it might additionally have an impact in the treatment of related diseases, such as ME/CFS.

The study is not without limitations. The number of controls was low (*n* = 28). However, the present analysis aimed to investigate differences within the post-COVID-19 syndrome cohort itself (*n* = 173). In addition, the age range for the present cohort was 39.7 ± 12 years. Cross-sectional studies are necessary with wider age-distribution ranges. In addition, it would be of interest if long-term studies could observe further alterations in OCT-A data over time. 

## 4. Material and Methods

### 4.1. Participants

One hundred and seventy three patients with post-COVID-19 syndrome (age: 39.7 ± 12; gender: 109 female, 64 male) and 28 controls (age: 29.2 ± 12; gender: 20 female, 8 male) were recruited at the Department of Ophthalmology, University of Erlangen-Nürnberg, Friedrich-Alexander-Universität Erlangen-Nürnberg (FAU). Post-COVID-19 syndrome was defined as the persistence of symptoms for more than 12 weeks after infection with the virus according to the S1 Guideline [6].

SARS-CoV-2 infection was confirmed by a positive real-time reverse transcription polymerase chain reaction test. Post-COVID-19 symptom persistency was 231 ± 111 days at the time of study participation. The most common self-reported post-COVID-19 symptoms in the present cohort were CF (92%), impaired concentration (83%), hair loss (63%), POTS (19%) and subjectively colder hands (12%). No local or systemic eye disorders with retinal affection were presented. The patients underwent ophthalmic examinations, including measurement of best-cured visual acuity (BCVA), non-contact intraocular pressure (IOP), measurement of axial length (IOL Master, Zeiss, Oberkochen, Germany) and OCT angiography (see below for details). Best-corrected visual acuity was 0.97 ± 0.1 (post-COVID-19 patients) and 1.12 ± 0.2 (controls). Anamnestic data, including self-reported chronic fatigue, were recorded. In addition, a subgroup of patients with post-COVID-19 assessed their self-reported fatigue using a chronic fatigue score system (Bell’s score; *n* = 104). The Bell’s score result was 46 ± 19. All patients signed a written informed consent form. The study was approved by the local ethics committee and performed in accordance with the tenets of the Declaration of Helsinki. 

### 4.2. GPCR-AAbs

A cardiomyocyte bioassay was used to analyze the presence and function of GPCR-AAbs [57,58,59]. Cardiomyocytes from newborn Wistar rats were transferred to a cardiomyocyte cell culture. In order to obtain GPCR-AAbs containing IgG, the patients’ sera were dialyzed against a dialyzing buffer of 0.15 M NaCl (10 mM phosphate buffer, pH of 7.4; Membra-Cel MD 44, 14 kDa, Serva) and stored at −20°C prior to analysis. This dialysate (40 µL) was added to the bioassay for 60 min. The basal beating rate of the cardiomyocytes was 1.8 beats/15 s. Any changes to this beating rate were measured: increases in the beating rate (i.e., positive chronotropy) or decreases in the beating rate (i.e., negative chronotropy). Positive chronotropic effects were measured for AT1-AAb, β2-AAb, α1-AAb and Noci-AAb. Negative chronotropic effects were measured for ETA-AAb, M2-AAb and MAS-AAb. Specification of the GPCR-AAb subtype was revealed by specific blockers, which were added to the bioassay: 1 μM of A779 (MAS-AAb), 0.1 μM of ICI118,551 (β2-AAb), 1 µM of atropin (M2-AAb), 0.1 μM J113397 (Noci-AAb), 1 µM losartan (AT1-AAb), 0. 1 μM BQ123 (ETA-AAb) and 1 μM urapidil or prazosin (α1-AAb). 

### 4.3. OCT-A

OCT-A (Heidelberg Spectralis II, Heidelberg, Germany) is a diagnostic technique used to visualize retinal microcirculation in the macula and peripapillary region. Retinal macula microvasculature can be subdivided into three layers: the superficial vascular plexus (SVP), the intermediate capillary plexus (ICP) and the deep capillary plexus (DCP). All OCT-A scans have an angle of 15° covering a size of 2.9 mm × 2.9 mm, with a lateral resolution of 5.7 µm/pixel [5]. 

The OCT-A data were exported by the SP-X1902 software (prototype software, Heidelberg Engineering, Heidelberg, Germany) and analyzed by the Erlangen-Angio-Tool (EA-Tool) software, which is coded in MATLAB (The MathWorks, Inc., Natick, MA, USA, R2017b). Studies have revealed high levels of reliability and reproducibility for the EA-Tool [60]. For the analysis, VD was computed in 12 sectors of the macula. Moreover, overall VD was computed as a mean over the sectors. In addition, the Anatomic Positioning System (APS—part of Glaucoma Module Premium Edition (GMPE), Heidelberg Engineering, Heidelberg, Germany) was implemented in the EA-Tool. This feature aligns all OCT-A scans according to their individual fovea-to-Bruch’s membrane opening center axis (FoBMOC) to allow for a better comparison of different scans. This FoBMOC axis is defined by the fovea and the center of Bruch’s membrane opening [5].

### 4.4. Statistical Analysis

The data were analyzed using different mixed models (SAS version 9.4, Institute Inc., Cary, NC, USA), taking into consideration the repetitions for the eyes (12 times) for each sector of the macula in the OCT-A scans. In the first model, we compared the PCS and the control patients; the variable was set as the independent variable. In the second model, we excluded the control group; the independent variable was chronic fatigue. We estimated the least square means (LS means) that corresponded to the specified effects for the linear predictor part of the model and the relative confidence limits. LS means are closer to reality and constitute more accurate data when cofactors occur compared to means. Age and gender were introduced as covariates in both models. In the second model, we also added Bell’s score as predicative variables. A mixed model was calculated also for the combination of the GPCR-AAb variables, testing possible differences in SVP, ICP and DCP. The *p*-values (the α-value was set at 0.05) are presented with their respective confidence interval limits (CLs). All the CLs and *p*-values in the multiple comparisons were adjusted with Tukey–Kramer tests. 

## 5. Conclusions

Post-COVID-19 syndrome is a post-infectious disease with a multifactorial pathomechanism and symptoms. We were able to reveal differences in VDs in the ICPs of the control and PCS patients. Considering the PCS patient group, we were able to observe differences in VD in the SVP between PCS patients with and without CF. In addition, GPCR-AAb showed an impact on impaired retinal microcirculation. As self-reported fatigue is one of the most common symptoms in PCS, the present study showed that vessel density in retinal microcirculation as measured by OCT-A might serve as an objective biomarker for this subjectively reported symptom. 

## Figures and Tables

**Table 1:** Differences in least square means of vessel densities in the SVP, ICP and DCP in patients with post-COVID-19 syndrome (PCS, 1) compared to controls. SVP—superficial vascular plexus, ICP—intermediate capillary plexus, DCP—deep capillary plexus.

Differences in Least Squares Means of Vessel Densities in Patients with PCS Compared to Controls									
	Effect	Controls	_PCS	Estimate	SE	*t*-Value	Pr > |t|	Lower	Upper
SVP	Post-COVID-19	0	1	0.16	0.2	0.81	0.42	−0.23	0.55
ICP	Post-COVID-19	0	1	0.66	0.2	3.84	0.0001	0.32	0.1
DCP	Post-COVID-19	0	1	0.11	0.2	0.54	0.59	−0.2855	0.5

**Table 2:** Differences in least square means of VD in the SVP, ICP and DCP in patients with post-COVID-19 syndrome (PCS) with chronic fatigue (CF, 1) and without CF (0). A notable significant effect was observed in the SVP (*p* = 0.0033).

Differences in Least Square Means of Vessel Density in PCS patients with CF (1) and without CF (0)									
	Effect	CF	_CF	Estimate	SE	*t*-Value	Pr > |t|	Lower	Upper
SVP	Chronic Fatigue	0	1	−2.71	0.91	−2.98	0.0033	−4.51	−0.92
ICP	Chronic Fatigue	0	1	−0.31	0.84	−0.37	0.71	−1.97	1.35
DCP	Chronic Fatigue	0	1	0.61	0.8	0.76	0.45	−0.97	2.19

**Table 3:** Differences in the least square means of VDs in the SVP, ICP and DCP in patients with post-COVID-19 syndrome (PCS), considering seropositivity for GPCR-AAb (1) and seronegativity for GPCR-AAb (0). Notable significant effects were observed in the SVP for β2-AAb (*p* = 0.0075) and in the ICP for Noci-AAb (*p* = 0.0344), β2-AAb (*p* = 0.0112) and ETA-AAb (*p* = 0.0261), and in the DCP an associative trend with Noci-AAb (*p* = 0.055) was also observed.

Differences in Least Square Means of Vessel Density in the SVP in PCS Patients Considering GPCR-AAb										
Effect	GPCR-AAb (0)	GPCR-AAb (1)	Estimate	SE	*t*-Value	Pr > |t|	Lower	Upper	Adj. Lower	Adj. Upper
Noci-AAb	0	1	0.69	0.44	1.58	0.1146	−0.17	1.55	−0.17	1.55
β2-AAb	0	1	−1.85	0.69	−2.7	0.0075	−3.20	−0.50	−3.20	−0.50
AT1-AAb	0	1	0.75	0.87	0.86	0.3914	−0.97	2.46	−0.97	2.46
α1-AAb	0	1	0.15	0.36	0.42	0.678	−0.55	0.85	−0.55	0.85
MAS-AAb	0	1	−0.42	0.91	−0.46	0.6439	−2.20	1.37	−2.20	1.37
M2-AAb	0	1	0.61	0.46	1.33	0.1833	−0.29	1.52	−0.29	1.52
ETA-AAb	0	1	−0.03	0.91	−0.03	0.9764	−1.82	1.77	−1.82	1.77
**Differences in Least Square Means of Vessel Density in the ICP in PCS Patients Considering GPCR-AAb**										
**Effect**	**GPCR-AAb (0)**	**GPCR-AAb (1)**	**Estimate**	**SE**	***t*** **-Value**	**Pr > |t|**	**Lower**	**Upper**	**Adj. Lower**	**Adj. Upper**
Noci-AAb	0	1	0.88	0.41	2.13	0.0344	0.07	1.70	0.07	1.70
β2-AAb	0	1	−1.66	0.65	−2.56	0.0112	−2.94	−0.38	−2.94	−0.38
AT1-AAb	0	1	1.27	0.90	1.4	0.1624	−0.51	3.05	−0.51	3.05
α1-AAb	0	1	0.36	0.34	1.07	0.2848	−0.30	1.03	−0.30	1.03
MAS-AAb	0	1	−0.85	0.92	−0.93	0.3553	−2.67	0.96	−2.67	0.96
M2-AAb	0	1	0.58	0.44	1.34	0.1826	−0.28	1.44	−0.28	1.44
ETA-AAb	0	1	−1.93	0.86	−2.24	0.0261	−3.63	−0.23	−3.63	−0.23
**Differences in Least Square Means of Vessel Density in the DCP in PCS Patients Considering GPCR-AAb**										
**Effect**	**GPCR-AAb (0)**	**GPCR-AAb (1)**	**Estimate**	**SE**	***t*** **-Value**	**Pr > |t|**	**Lower**	**Upper**	**Adj. Lower**	**Adj. Upper**
Noci-AAb	0	1	0.87	0.45	1.93	0.055	−0.02	1.75	−0.02	1.75
β2-AAb	0	1	−1.08	0.70	−1.53	0.1271	−2.47	0.31	−2.47	0.31
AT1-AAb	0	1	0.50	0.89	0.56	0.5788	−1.26	2.26	−1.26	2.26
α1-AAb	0	1	−0.09	0.37	−0.26	0.7973	−0.81	0.63	−0.81	0.63
MAS-AAb	0	1	−0.16	0.93	−0.18	0.8595	−2.00	1.67	−2.00	1.67
M2-AAb	0	1	0.40	0.47	0.85	0.3976	−0.53	1.33	−0.53	1.33
ETA-AAb	0	1	−1.83	0.94	−1.96	0.0516	−3.67	0.01	−3.67	0.01

## Data Availability

The original contributions presented in the study are included in the article.
